# Molecular Cytogenetic and Physiological Characterization of a Novel Wheat-Rye T1RS.1BL Translocation Line from *Secale cereal* L. Weining with Resistance to Stripe Rust and Functional “Stay Green” Trait

**DOI:** 10.3390/ijms23094626

**Published:** 2022-04-21

**Authors:** Zhi Li, Qing Jiang, Tao Fan, Liqi Zhao, Zhenglong Ren, Feiquan Tan, Peigao Luo, Tianheng Ren

**Affiliations:** 1 College of Agronomy, Sichuan Agricultural University, Wenjiang, Chengdu 611130, China; lizhi@sicau.edu.cn (Z.L.); j15982073347@163.com (Q.J.); 18328080816@163.com (T.F.); lq999624001@163.com (L.Z.); renzllab@sicau.edu.cn (Z.R.); feiquantan_1@163.com (F.T.); lpglab@sicau.edu.cn (P.L.); 2 Provincial Key Laboratory for Plant Genetics and Breeding, Wenjiang, Chengdu 611130, China

**Keywords:** wheat, chromosome translocation, resistance, leaf senescence, Weining rye, FISH

## Abstract

In this study, a novel T1RS.1BL translocation line RT843-5 was selected from a cross between wheat Mianyang11 (MY11) and Weining rye. The results of MC-FISH, PCR, and A-PAGE showed that RT843-5 contained two intact T1RS.1BL translocation chromosomes. RT843-5 showed resistance to the most virulent and frequently occurring stripe rust races/isolates. Additionally, RT843-5 showed resistance in the field in locations where stripe rust outbreaks have been the most severe in China. Genetic analysis indicated one new gene for stripe rust resistance, located on 1RS of RT843-5, which was tentatively named *YrRt843*. Furthermore, the chlorophyll content, the activities of catalase (CAT), and superoxide dismutase (SOD), and the net photosynthetic rate (Pn) of RT843-5 were significantly higher than those in its wheat parent MY11, whereas malondialdehyde (MDA) accumulation was significantly lower after anthesis in RT843-5 compared to in MY11. RT843-5 had a significantly higher 1000-kernel weight and yield than MY11. The results indicated that RT843-5 exhibited functional stay-green traits after anthesis, that delayed the senescence process in wheat leaves during the filling stage and had positive effects on grain yield. The present study indicated that Weining rye may carry untapped variations as a potential source of resistance, and that RT843-5 could be an important material for wheat breeding programs in the future.

## 1. Introduction

Wheat (*Triticum aestivum* L.) is one of the most important crops in the world. Stripe rust, which is caused by *Puccinia striiformis* Westend. f. sp. *tritici* ERikss. (*Pst*), is considered one of the most devastating diseases in wheat. Introducing an alien chromosome into the wheat genome is an efficient approach to enhance the genetic diversity and disease resistance ability of wheat [1,2,3]. Many resistance genes against stripe rust have been introduced into wheat from different related species, such as *Yr50* and *Yr69* from *Thinopyrum* spp. [4,5] and *Yr17*, *Yr37*, *Yr38*, and *Yr40* from *Aegilops* spp. [6,7,8,9], and many other from *Leymus mollis*, *Psathyrostachys huashanica*, etc. [10,11]. However, the most important and successful relative species used in wheat breeding programs is rye (*Secale cereal* L.) [12,13,14]. Through the T1RS.1BL translocation, the resistance genes *Yr9*, *Pm8*, *Lr26*, and *Sr31* and several genetic factors could enhance the yield and environmental adaptability upon introduction into the wheat genome [15,16,17]. Therefore, T1RS.1BL translocation has been widely used in wheat breeding programs worldwide during the last century [13]. Unfortunately, since the 1990s, the 1RS chromosome arm which is a single-origin arm from the German rye “Petkus” [12,14,18], and changes in the prevalence of virulent pathogens, the resistance genes located on Petkus’ 1RS chromosome have been overcome [14,19]. However, due to the great advantage of the high yield with 1RS.1BL translocations, it is still used in many countries’ wheat breeding programs. For example, 539 out of 1293 wheat cultivars (lines) in China in 2020 still contained 1RS.1BL translocation chromosomes [20].

The leaf senescence process after anthesis is important and closely related to biological yield [21]. A specific trait of the leaf senescence process is called “stay-green”, defined as the delayed deconstruction of the photosynthetic apparatus during leaf senescence [21,22]. The stay-green trait has been divided into two types: functional stay-green and nonfunctional stay-green [21]. To date, there have been several reports of stay-green traits in sorghum (*Sorghum bicolor*) [23], rice (*Oryza sativa*) [24,25], soybean (*Glycine max*) [26], maize (*Zea mays*) [27], rapeseed (*Brassica napus*) [28], tobacco (*Nicotiana tabacum*) [29], sweet potato (*Dioscorea esculenta*) [30], and wheat (*Triticum turgidum* and *Triticum aestivum*) [31,32,33,34]. Only the functional stay-green trait can prolong photosynthesis and the duration of grain filling [21]. However, many reported stay-green traits are nonfunctional, which means that the leaves of stay-green plants remain green, but the photosynthetic capacity is lost [32,34,35]. Several genes were also indicated to be related to stay-green traits, such as *PAO* (*pheophorbide a oxygenase*), *NYC1* (*NON-YELLOW COLORING 1*), *NOL* (*NYC1-LIKE*), and *SGR/SGRL* (*STAYGREEN/SGR-LIKE*) [36], however, most of these genes were non-functional [36]. On the other hand, functional stay-green plants are rare in nature [35]. For example, Spano et al. [32] reported four functional stay-green durum wheat lines, which were induced by ethylmethane sulfonate (EMS) and exhibited better net photosynthetic rates than the parental plants. Wang et al. [34] reported a wheat stay-green mutant *tasg1* (also induced by EMS), in which cytokinin metabolism was involved in delayed flag leaf senescence.

The elite rye variety Weining was used as the plant material for rye genome sequencing in the previous study [37]. A total of 39,355 genes were identified in the Weining rye genome, and, among them, 1909 disease resistance-associated genes were mapped to the seven assembled chromosomes of Weining rye [37]. Weining rye is thought to have a large number of untapped resistance genes that could be useful in wheat breeding programs. In the present study, a novel T1RS.1BL translocation line, named RT843-5, whose 1RS chromosome arms were from the Weining rye, was developed. RT843-5 exhibited resistance to stripe rust in both the seedling and adult stages. Genetic analysis also indicated that the stripe rust resistance gene in the 1RS chromosome of RT843-5 was different from *Yr9*. Therefore, it was presumed that a new resistance gene, tentatively named *YrRt843,* was located on the 1RS chromosome of RT843-5. Moreover, RT843-5 exhibited a functional stay-green trait and high yield when compared with its wheat parent MY11. This translocation line could be a very important genetic resource for wheat breeding programs in the future.

## 2. Results

### 2.1. Development of the New T1RS.1BL Translocation Line RT843-5

Weining rye (2*n* = 14, RR) and the wheat parent Mianyang11 (MY11) (2*n* = 42, AABBDD) were crossed directly. The amphidiploid plants (2*n* = 56, AABBDDRR) were backcrossed twice with MY11. A 1R monosomic addition plant (2*n* = 43, 21″WW + 1′1R) was selected from the BC_2_F_2_ generation by multiple-color fluorescence in situ hybridization (MC-FISH). This plant exhibited high resistance to stripe rust and was named 98–843. Then, the offspring of 98–843 were reproduced by continued selfing. Only the plants that showed resistance were sequentially chosen and reproduced to obtain the next generation. In the BC_2_F_5_ generation (F_3_ of 98–843), 30 seeds were detected by MC-FISH before sowing, and one plant was identified to contain a pair of 1RS.1BL translocation chromosomes and exhibited resistance to stripe rust and stay-green traits in the field.

### 2.2. Chromosome Identification

**Figure 1:**
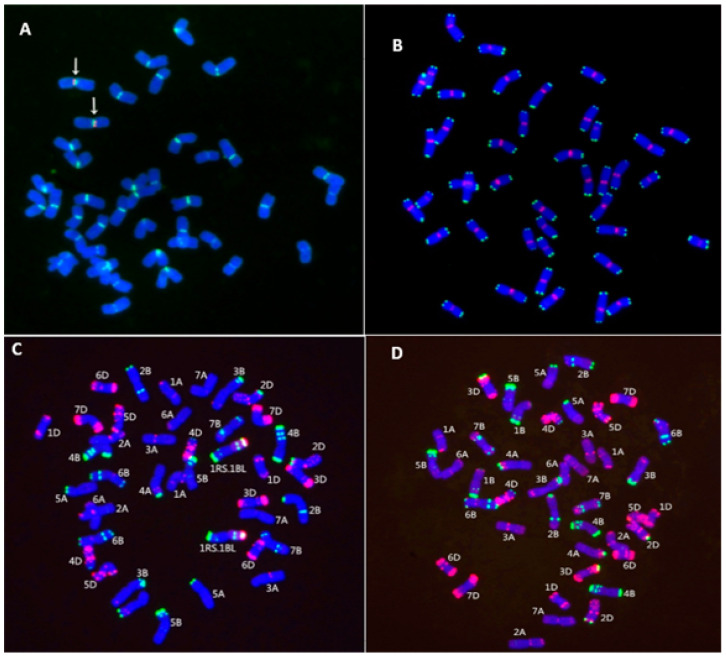
Chromosome identification of RT843-5 by MC-FISH. (**A**): Signal patterns of pMD-CEN-3 (a rye-specific centromeric probe, red) and 6c6 (a wheat-specific centromeric probe, green) joined together in the centromere region. The arrows indicate the translocation chromosomes. (**B**): 6c6: red, (5′-CCCTAAA-3′)_4_ (a telomere-specific probe): green. (**C**): Chromosome constitution of the novel T1RS.1BL translocation line RT843-5 (2*n* = 42). (**D**): Chromosome structure of MY11. No rye chromatin was detected.

**Figure 2:**
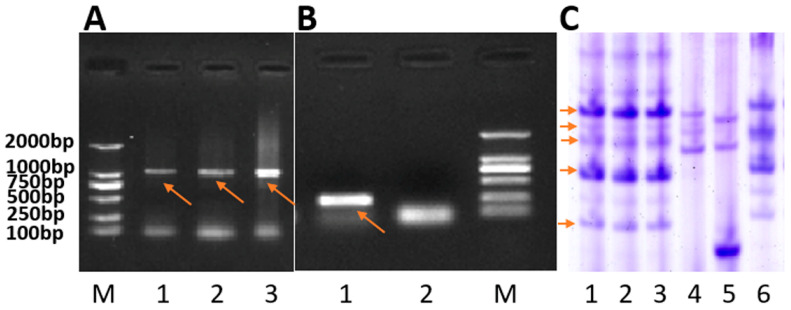
Chromosome identification of RT843-5 by PCR and A-PAGE. (**A**): PCR results obtained with the primer pair ω-Sec-P. Lanes 1 to 3: different RT843-5 plants; Lane M: DNA marker DL2000. The arrows show the specific bands amplified by ω-Sec-P. (**B**): PCR results obtained with the primer pair Gil-B1. Lane 1: MY11; lane 2: RT843-5; and lane M: DNA marker DL2000. The arrows show the specific bands amplified by Gil-B1. (**C**): A-PAGE separations of ω-secalin from RT843-5. Lanes 1 to 3: different seeds from RT843-5; lane 4: Chinese Spring; lane 5: MY11; lane 6: CN11. The arrows show the ω-secalin proteins encoded by the *Sec-1* gene.

In the genome of wheat parent MY11, no rye chromatin was found (Figure 1D). On the other hand, in RT843-5, the signal of the wheat-specific centromeric probe 6c6 was combined with the signal of the rye-specific centromeric probe pMD-CEN-3 (Figure 1A). The signals of the telomere-specific probe (CCCTAAA)_4_ and the wheat-specific centromeric probe 6c6 were both present on all chromosomes of R843-5 (Figure 1A,B). The probes of rye genomic, pSc119.2 and pAs1 showed that RT843 contained a pair of 1RS.1BL translocation chromosomes (Figure 1C). The analysis of MC-FISH indicated that RT843-5 (2*n* = 42) harbored a pair of intact T1RS.1BL translocation chromosomes (Figure 1A–C), and the 1RS chromosome arms were from Weining rye. The primer pair ω-Sec-P amplified a 1076 bp band from the rye 1RS chromosome arm, and the primer pair Gil-B1 amplified a 220 bp band from the wheat 1BS chromosome arm. The presence of 1RS chromosome arm was confirmed in the 1RS.1BL translocation line RT843-5, as indicated by the presence of 1076 bp band, while the presence of 1BS of wheat was confirmed in the wheat parent MY11 (the presence of 220 bp band) (Figure 2A,B). The results suggested that the wheat 1BS chromosome arms of RT843-5 were replaced by rye 1RS chromosome arms. The specific protein bands of ω-secalin were detected by acid polyacrylamide gel electrophoresis (A-PAGE). The control CN11 was a 1RS.1BL translocation, and the ω-secalin of CN11 would be expressed in the seeds. The specific bands of ω-secalin of 1RS.1BL translocation lines could be detected in the ω-area of the A-PAGE patterns [18]. Therefore, RT843-5 would have similar patterns of proteins to CN11 in the ω-area in A-PAGE. RT843-5 samples exhibited the expression of genes at the *Sec-1* locus on the 1RS chromosome arm (Figure 2C). These results confirmed that RT843-5 was a new T1RS.1BL translocation line with a pair of 1RS chromosomes from Weining rye.

### 2.3. Analysis of Resistance to Stripe Rust

**Figure 3:**
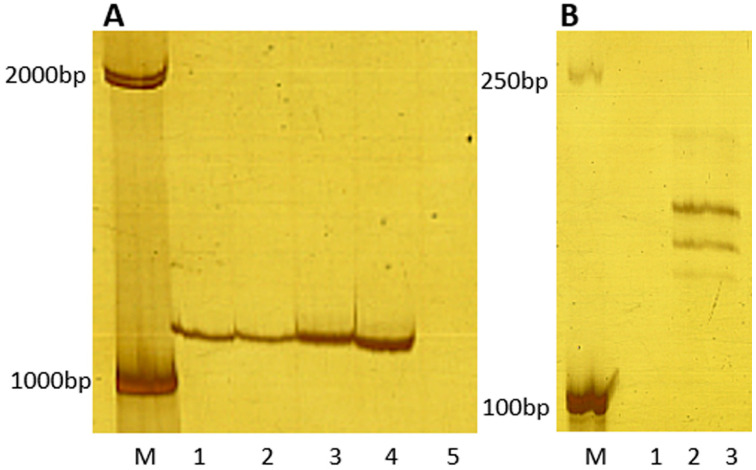
PCR results from primers Iag95 and Xgwm582. (**A**): PCR results of Iag95. Lane 1: Kavkaz (Yr9); lane 2: Aurora (Yr9); lane 3: RT843-5; lane 4: RT843-5; lane 5: negative control (ddH_2_O for template). (**B**): PCR results of Xgwm582. Lane 1: negative control (ddH_2_O for template), lane 2: Aurora (Yr9); lane 3: RT843-5.

All lines/cultivars were examined for resistance to stripe rust induced by eight *Pst* races/isolates (CYR32, CYR33, CYR34, SY3, SY4, SY5, SY7, and HY8) in the greenhouse during the seedling stages. The wheat parent MY11 was susceptible to all *Pst* races/isolates except HY8. CN11, which inherited its 1RS chromosomes from Petkus rye and contained the *Yr9* gene, was susceptible to all *Pst* races/isolates except SY5. RT843-5, which was developed and identified in this study, showed resistance against all *Pst* races/isolates that were used in this study (Table 1). The lines/cultivars were also grown in the field under severe natural *Pst* infection at the Qionglai Research Station of Sichuan Agricultural University in Southwest China. Field experiments also indicated that MY11 and CN11 were susceptible to stripe rust. On the other hand, RT843-5 showed resistance to stripe rust in the field (Table 1). The results suggested that the new T1RS.1BL translocation line RT843-5 displayed seedling and adult resistance to stripe rust. In addition, the testcross populations of RT843-5 × MY11 ((RT843-5 × MY11) × MY11) exhibited monogenic segregation (1 resistant: 1 susceptible, χ^2^ = 0.62) when tested with mixed *Pst* races/isolates (Table 2). Segregation in the RT843-5 × MY11-derived F_2_ population also conformed to a 3:1 resistance to susceptibility ratio (χ^2^ = 0.60) when tested with mixed *Pst* races/isolates (Table 2). The F_2_-susceptible plants (67 plants) continued to exhibit susceptibility to *Pst* in the F_3_ population. A total of 160 of 224 lines that were resistant to *Pst* in F_2_ exhibited resistance/susceptible segregation in the F_3_ population. The F_3_ population exhibited monogenic segregation (64 resistant: 160 segregations: 67 susceptible, 1 homozygote: 2 heterozygous: 1 homozygote, χ^2^ = 2.95). Furthermore, two closely linked *Yr9* molecular markers Xgwn582 and Iag95 were used to detect the *Yr9* gene in RT843-5. The PCR results showed that the same bands were amplified in Kavkaz and Aurora (both carrying *Yr9*), as well as in RT843-5 (Figure 3), which suggested that RT843-5 might also carry the *Yr9* gene. However, RT843-5 showed high resistance to stripe rust. These results indicated the presence of a single dominant gene, different from *Yr9*, in RT843-5 resistance to stripe rust was tentatively named *YrRt843*.

### 2.4. Measurement and Analysis of Functional Stay-Green Traits

**Figure 4:**
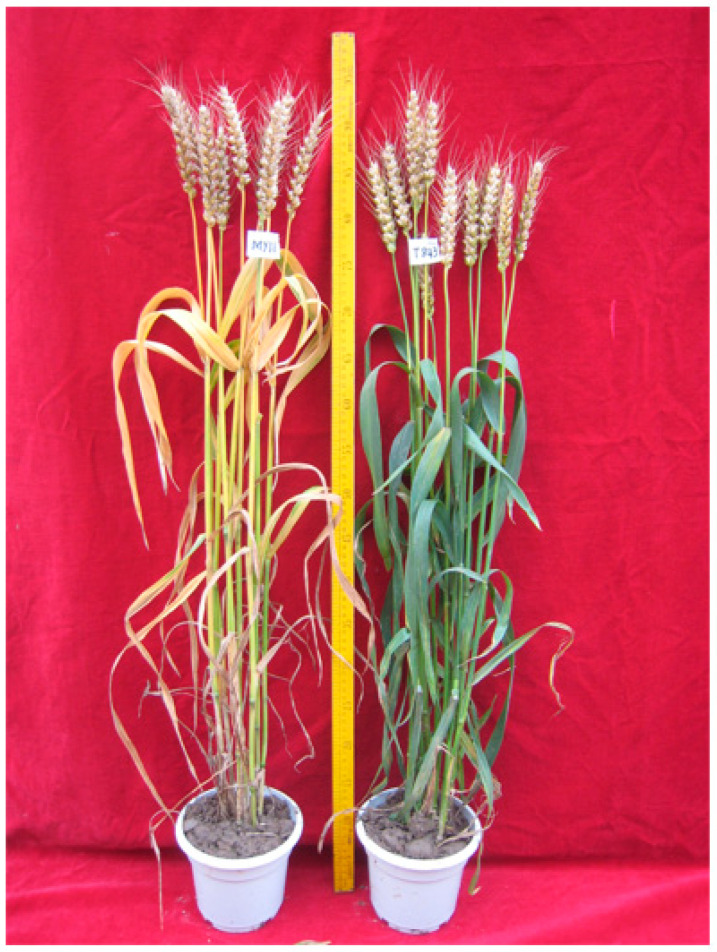
RT843-5 exhibited stay-green traits during the filling period. **Left:** wheat parent, nonstay- green line MY11. **Right:** RT843-5.

RT843-5 exhibited the stay-green trait during the filling period (Figure 4). To confirm whether this trait was functional, we carried out the following experiment.

**Figure 5:**
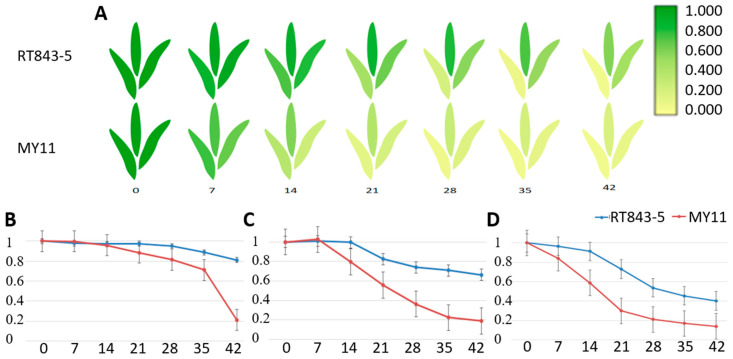
Differences in the SGI (stay-green index) between RT843-5 and MY11 during leaf senescence after anthesis. (**A**): Diagram of the changes in the SGI in the FL (flag leaf), SL (second leaf), and TL (third leaf) at 0, 7, 14, 21, 28, 35, and 42 days after anthesis. (**B**): The change curve of the SGI of the FL after anthesis. (**C**): The change curve of the SGI of the SL after anthesis. (**D**): The change curve of the SGI of the TL after anthesis. The Y-axis shows the values of the SGI, and the numbers on the X-axis show the days after anthesis.

The chlorophyll contents of the FL (flag leaf), SL (second leaf), and TL (third leaf) were measured, and then the SGI (stay-green index) was calculated. The results showed that after anthesis, the leaves of RT843-5 and MY11 rapidly entered the senescence process. However, the speed of the senescence process was obviously significantly different between stay-green line RT843-5 and nonstay-green line MY11 (Table S1, Figure 5). The decrease curves of FL, SL, and TL showed that the decrease in the SGI of stay-green line RT843-5 was significantly slower than that of nonstay-green line MY11, and the SGI of RT843-5 (FL: 0.813, SL: 0.662, TL: 0.401) at 42 DAA (days after anthesis) was still significantly higher (*p* < 0.05) than that of MY11 (FL: 0.212, SL:0.191, TL: 0.139) (Table S1, Figure 5).

**Figure 6:**
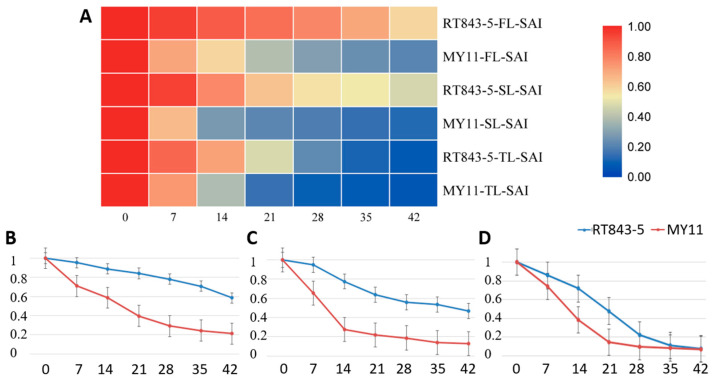
Differences in the SAI (SOD activity index) between RT843-5 and MY11 during leaf senescence after anthesis. (**A**): Heatmap of the changes in the SAI in the FL, SL, and TL. The different colors show the different values of SAI. (**B**): The change curve of the SAI of the FL; (**C**): The change curve of the SAI of the SL; (**D**): The change curve of the SAI of the TL. The Y-axis shows the SAI values, and the numbers on the X-axis show the days after anthesis.

The decrease curves of the SAI (SOD activity index) were similar to the SGI decrease curve in the FL and SL, but the SAI of the TL was different (Table S2, Figure 6). The SAI of the TL of RT843-5 was significantly higher (*p* < 0.05) than that of MY11, except at 42 DDA (RT843-5: 0.021, MY11: 0.068), during the leaf senescence process (Table S2, Figure 6). On the other hand, the SAIs of the FL and SL of RT843-5 were always significantly higher (*p* < 0.05) than those of MY11 during the leaf senescence process (Table S2, Figure 6), and the SGI of RT843-5 (FL: 0.585, SL: 0.469) at 42 DAA was still significantly higher (*p* < 0.05) than that of MY11 (FL: 0.210, SL: 0.126).

**Figure 7:**
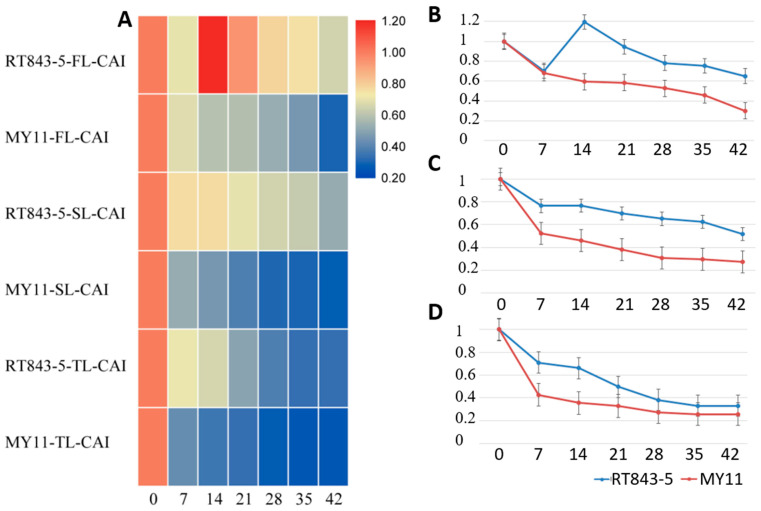
Differences in the CAI (CAT activity index) between RT843-5 and MY11 during leaf senescence after anthesis. (**A**): Heatmap of the changes in the CAI in the FL, SL, and TL. The different colors show the different values of the CAI. (**B**): The change curve of the CAI of the FL; (**C**): The change curve of the CAI of the SL; (**D**): The change curve of the CAI of the TL. The Y-axis shows the CAI values, and the numbers on the X-axis show the days after anthesis.

The decrease curves of the CAI (CAT activity index) were very different from the curves of the SGI and SAI (Table S3, Figure 7). After anthesis, the CAI of the FL of both RT843-5 (0.704) and MY11 (0.683) decreased in the first 7 days. Then, the CAI of the stay-green line RT843-5 increased to 1.195, while the CAI of the nonstay-green line MY11 continued to decrease to 0.593 at 14 DAA (Table S3, Figure 7). The CAI of the FL of RT843-5 at 14 DAA suggested that at this time, the CAT activity was strongest during the senescence process in the stay-green plants. Finally, the CAI of the FL of RT843-5 (0.651) was significantly higher (*p* < 0.05) than that of MY11 (0.299) at 42 DAA (Table S3, Figure 7). The CAIs of the SL and TL continued to decrease during the senescence process in both RT843-5 and MY11 (Table S3, Figure 7). However, the CAI values of the SL (0.519) and TL (0.331) of RT843-5 were significantly higher (*p* < 0.05) than those of MY11 (0.275 and 0.258, respectively) at 42 DAA. (Table S3, Figure 7).

**Figure 8:**
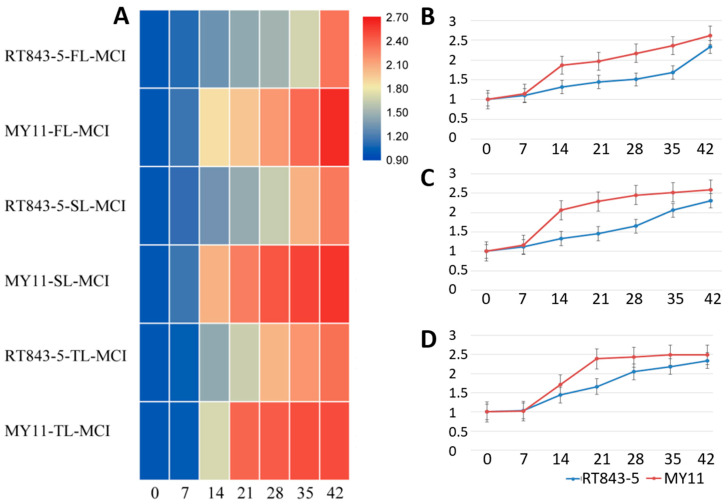
Differences in the MCI (MDA content index) between RT843-5 and MY11 during leaf senescence after anthesis. (**A**): Heatmap of the changes in the MCI in the FL, SL, and TL. The different colors show the different values of MCI. (**B**): The change curve of the MCI of the FL; (**C**): The change curve of the MCI of the SL; (**D**): The change curve of the MCI of the TL. The Y-axis shows the values of the MCI, and the numbers on the X-axis show the days after anthesis.

The MCI (MDA content index) of both RT843-5 and MY11 increased after anthesis (Table S4, Figure 8). After 7 to 21 DAA, the MCI of the nonstay-green line MY11 was drastically altered. At 14 DAA, for example, the MCI of the FL of MY11 was up to 1.865. However, when compared to MY11, the MCI of RT843-5 was altered significantly more smoothly. For example, at 14 DAA, the MCI of the FL of RT843-5 was only up to 1.318. Furthermore, at any moment after 14 DAA, the MCI of RT843-5 was significantly lower (*p* < 0.05) than that of MY11 (Table S4, Figure 8). Finally, the MCIs of the FL, SL, and TL of RT843-5 were 2.330, 2.312, and 2.344 at 42 DAA, respectively. The MCIs of FL, SL, and TL of MY11 at 42 DAA were 2.627, 2.591, and 2.491, respectively (Table S4, Figure 8). The MCI of the stay-green line RT843-5 was significantly lower (*p* < 0.05) than that of the nonstay-green line MY11.

**Figure 9:**
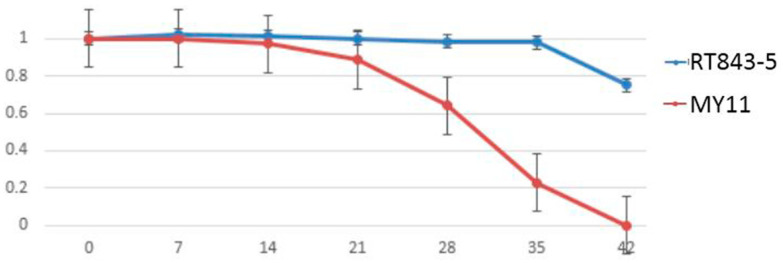
Differences in the NPI (net Pn index) between RT843-5 and MY11 during leaf senescence after anthesis. The Y-axis shows the values of the NPI, and the numbers on the X-axis show the days after anthesis.

The results of the NPI (net Pn index) indicated that RT843-5 exhibited high Pn (net photosynthetic rate) after anthesis, especially in the later filling period (Table S5, Figure 9). The NPI of MY11decreased tremendously at 21 DAA. The NPI of MY11 decreased from 0.889 to 0.643 from 21 DAA to 28 DAA and continued to decrease to 0.228 at 35 DAA. On the other hand, the NPI of RT843-5 decreased from 1.001 to 0.985 from 21 DAA to 28 DAA and continued to decrease to 0.981 at 35 DAA. At 42 DAA, the leaves of MY11 were too yellow and withered to measure Pn. In contrast, the leaves of RT843 remained green, and the NPI was 0.752, which was significantly better than that of MY11. These results indicated that RT843-5 still maintained a high photosynthetic rate at the late grain-filling stage (Table S5, Figure 9).

When compared to the nonstay-green line MY11, the findings regarding the SGI, SAI, CAI, MCI, and NPI suggested that the leaves of stay-green line RT843-5 exhibited a slow senescence process. Because of the greater enzyme activity and high Pn in the later grain filling period, all evidence suggested that RT843-5 is a functional stay-green line.

### 2.5. Agronomic Traits of RT843-5

Between RT843-5 and MY11, there were no significant differences in the number of spikes per square meter (NS), kernel number per spike (KN), above-ground biomass (AGB), and harvest index (HI) (Table 3). However, the yield and 1000-kernel weight (TKW) of stay-green line RT843-5 were significantly higher than in its wheat parent MY11 (Table 3). The yield and TKW of RT843-5 were higher than those of MY11 by 13.4% and 7.66%, respectively. The results indicated that the stay-green trait produced more carbohydrates during the later seed filling period. The results also indicated that the functional stay-green trait of RT843-5 had a positive effect on grain yield.

## 3. Discussion

### 3.1. New 1RS Chromosome Arm with a Resistance Gene to Pst Originating from Weining Rye

Several important genes were discovered on the rye 1RS chromosome arms in the previous studies [15,17,38]. Most of these genes were disease, pest, drought, and lodging resistance genes [1,15,39,40,41]. One of the most essential and beneficial genes on the 1RS chromosome arm is the *Yr9* gene, which confers resistance to stripe rust in wheat [1,19]. Unfortunately, due to the rapid changes in prevalent virulent pathogens, the resistance of the wheat with *Yr9* has been completely overcome [1,18,19]. However, rye is a cross-pollinated plant with high genetic diversity within populations of a given variety. It was suggested that there could be an abundance of useful genes hidden in the rye genomes of different varieties [42]. For example, the 1RS.1BL translocation line R14, which originated from Petkus, contained the stripe rust resistance genes *YrCn17* and *PmCn17*, different from *Yr9* and *Pm8* [14]. The 1RS.1AL translocation line, ‘Amigo,’ contained a 1RS chromosome from the rye ‘Insave’ and the powdery mildew resistance gene *Pm17* [43]. The 1RS chromosome arm of ‘Kriszta’ rye has been introduced into the wheat genome, conferring stripe rust resistance to common wheat, and the resistance was also different from *Yr9* [44]. Further exploration of different rye 1RS resources may lead to the discovery of a large number of different disease-resistant resources. Weining rye, which is an elite rye variety from Southwestern China, showed high resistance to stripe rust in the field [1,37]. The reference genome of Weining rye contains 1909 disease resistance-associated (DRA) genes, 242 of which are found on the 1R chromosome [37]. The DRA genes may facilitate efficient genetic investigations and lead to improvement of disease resistance in wheat, given the critical relevance of disease resistance-linked genes in plant responses to biotic adversities. Weining rye has also been highlighted as a viable rye resource for wheat disease resistance improvement [37]. In the present study, a novel T1RS.1BL translocation line, RT843-5, was developed from a cross between common wheat MY11 and Weining rye. In the field, under severe natural *Pst* infection, RT843-5 showed resistance to stripe rust, as well as resistance to the prevalent virulent *Pst* races/isolates occurring in China (Table 1). Because the wheat parent MY11 was highly susceptible to stripe rust, the new resistance genes of RT843-5 have a high probability of being located on 1RS chromosomes. The testcross ((RT843-5 × MY11) × MY11) population, F_2_ and F_3_ populations of RT843 × MY11 exhibited monogenic segregations when they were tested with a mixture of *Pst* races/isolates (Table 2). Because the resistance patterns of RT843-5 to stripe rust are completely different from those of the *Yr9* gene (Table 1), the single dominant resistance gene present in RT843-5 is distinct from *Yr9*. We tentatively named the stripe rust resistance gene carried by RT843-5 *YrRt843*. *YrRt843* could be a new stripe rust resistance gene located on the rye 1RS chromosome. The novel germplasm with the 1RS.1BL translocation chromosome and the new stripe rust resistance gene *YrRt843* could serve as a valuable genetic resource for wheat resistance improvement in the future.

### 3.2. Functional Stay-Green Trait of RT843-5

During grain filling, the top three leaves of wheat are the major producers of photosynthates, contributing to approximately 80% of the net photosynthesis in the entire plant [45]. As a result, delayed leaf senescence may affect the supply of nutrients to developing grains during grain filling. During leaf senescence, we measured several parameters in the FL, SL, and TL to determine whether there were any variations in the dynamic changes in these parameters between stay-green wheat (RT843-5) and nonstay-green wheat (MY11). Reactive oxygen species (ROS), such as H_2_O_2_ and $O_{2}^{-}$, are signaling molecules that have many functions in plant development, among others causing leaf senescence [46,47]. The rate of elimination of excess ROS in plants is a measurement of photosynthetic competence. During leaf senescence, higher SOD activity has been attributed to increased $O_{2}^{-}$ production [48,49]. As a result, high SOD activity is thought to be a defensive system against leaf senescence, as well as to provide stability to the photosynthetic apparatus [46]. In the present study, the FL, SL, and TL of RT843-5 exhibited significantly higher SOD activity than those of MY11 during the leaf senescence process (Table S2, Figure 6). These results suggested that RT843-5 had a much better capacity to eliminate $O_{2}^{-}$. CAT is thought to be the key element in protecting the plant against oxidative stress, because it eliminates excess H_2_O_2_ from leaves [47,50]. The FL of RT843-5 was shown to be more resistant to leaf senescence than MY11 from 7 to 14 DAA, and the SL and TL of RT843-5 were found to be more resistant to oxidative stress (Table S3, Figure 7). According to the findings, RT843-5 had significantly better ability to remove H_2_O_2_. Additionally, the MDA content is an important factor in determining the degree of general lipid peroxidation. As a result, MDA accumulation can be used as a metric for determining the degree of senescence in plant leaves [50,51]. The MDA content of RT843-5 after 7 DAA was significantly lower than that of MY11 (Table S4, Figure 8). These findings revealed that during leaf senescence after anthesis, excess oxidation did not occur in the leaves of RT843-5. Furthermore, when RT843-5 was compared to MY11, greatly reduced chlorophyll loss and significantly higher Pn during senescence process were observed (Tables S1 and S5, Figure 5 and Figure 9). The significant differences in the estimated values of CAT, SOD, MDA, chlorophyll, and Pn between RT843-5 and MY11 suggested that the photosynthetic apparatus activities in RT843-5 were maintained at a greater level throughout the grain filling stage. RT843-5 had a much greater TKW and yield than MY11 due to its good photosynthetic ability during the filling stage (Table 3). All of this evidence suggested that RT843-5 had a functional stay-green trait after anthesis, that effectively delayed senescence process in wheat leaves during the filling stage, and had beneficial effects on grain yield.

## 4. Materials and Methods

### 4.1. Plant Materials

MY11 is a widely used common wheat cultivar in southwestern China, that was released in the 1980s. MY11 has been very susceptible to stripe rust in southwest China since the 1990s [52]. The pure genetic stock of MY11 used in this study was bred by single spike descent over several generations. Because MY11 carries the *kr1* gene, it can be crossed directly with rye [52]. The elite rye variety “Weining” was used as a donor. The wheat receptor MY11 was crossed with Weining rye, and the procedure for the development of new translocation lines was performed according to the method of Ren et al. [52]. The 1RS.1BL translocation cultivar Chuannong11 (CN11), carrying a pair of 1RS.1BL translocation chromosomes with *Yr9* derived from rye Petkus, which was released in 2003, was used in the comparative disease response tests.

### 4.2. Cytogenetic and Molecular Analyses

MC-FISH was used to identify the chromosome constitution of RT843-5. Weining rye genomic DNA, *Aegilops tauschii* clone pAs1, and rye clone pSc119.2 were used as probes to distinguish wheat, rye, and translocation chromosomes on the same slide. pAs1 can distinguish the D genome and the 1A, 2A, 3A, 4A, 6A, 7A, 1B, 2B, 3B, 6B, and 7B chromosomes of wheat. pSc119.2 can distinguish the B genome, the 2A, 4A, 5A, 2D, 3D, and 4D chromosomes of wheat, and the R genome of rye [53]. As a result, the combination of probes pAs1, and pSc119.2 with rye genomic DNA can distinguish different wheat and rye chromosomes in one cell [53]. The Weining rye genomic DNA and pAs1 probe were labeled with Texas red-5-dUTP (Invitrogen) (red). The probe pSc119.2 was labeled with Alexa Fluor-488-5-dUTP (Invitrogen) (green). Clone 6c6, pMD-CEN-3, and sequence (5′-CCCTAAA-3′)_4_ were also used as probes to identify the centromeres and telomeres of chromosomes [18]. The sample preparation (root tips of wheat), probe labeling, in situ hybridization, and images captured were performed according to Du et al. [54].

PCR was also used to determine the chromosome constitution of RT843-5. Genomic DNA was isolated from young leaves by the surfactant cetyltrimethylammonium bromide method [55]. The primer pair ω-Sec-P was used to detect the 1RS of rye [56], and Gil-B1 to detect the 1BS of wheat (Table 4) [57]. PCR was performed according to Chai et al. [56] and Zhang et al. [57].

The *Sec-1* gene located on the 1RS chromosome arm encodes ω-secalin, which can be used as a marker for the presence of the 1RS chromosome [18]. The detection of ω-secalin proteins on A-PAGE was conducted as described by Ren et al. [18].

Molecular markers Xgwm582 and Iag95 were closely linked to *Yr9* (Table 4) [58,59]. These two molecular markers were used to determine the presence of the *Yr9* gene in RT843-5. PCR was performed according to Weng et al. [58] and Mago et al. [59].

### 4.3. Stripe Rust Tests

RT843-5 was examined for resistance to stripe rust induced by eight *Pst* races/isolates. This study included three prevalent *Pst* races in China, CYR32, CYR33, and CYR34, and five prevalent *Pst* isolates, SY3, SY4, SY5, SY7, and SY8. The *Pst* races/isolates were provided by the Plant Protection Institute, Gansu Academy of Agricultural Sciences, China. CYR32 is virulent to *Yr1*, *2*, *3*, *4*, *6*, *7*, *8*, *9*, *17*, *25*, *27*, *28*, *31*, *32*, *43*, *44*, *A*, *Alba*, *Cle*, *Gaby*, *Res*, *SD*, *SO*, *Exp2*, *SK*, and *SP*. CYR33 is virulent to *Yr1*, *2*, *3*, *4*, *6*, *7*, *8*, *9*, *17*, *25*, *28*, *31*, *32*, *A*, and *Su.* CYR34 is virulent to virulent t *Yr1*, *2*, *3*, *4*, *6*, *7*, *8*, *9*, *10*, *17*, *19*, *24* (=*26*), *25*, *27*, *28*, *31*, *32*, *43*, *44*, *Exp2*, *SP*, *A*, and *Sk* [52,60]. *Pst* isolates SY3, SY4, SY5, SY7, and HY8 have been virulent to many released wheat cultivars in China in the recent decades and are to *Yr9* [18]. Three seedlings of RT843-5 at the two-leaf stage were inoculated under controlled conditions in the greenhouse, according to the method described by Ren et al. [1] and Wan et al. [61], with three replications. RT843-5 was also grown in the field under severe natural *Pst* infection at the Qionglai Research Station of Sichuan Agricultural University in Southwest China (in Chengdu Plain, 30°25′ N, 103°28′ E, 493.3 m above sea level).

RT843-5 was backcrossed with MY11. The testcross (418 plants) and the F_2:3_ populations of RT843-5 × MY11 (291 lines) were used to determine the chromosomal location of the gene against stripe rust. The wheat parent MY11 and wheat cultivar CN11 (containing *Yr9*) were used as controls. Infection types (IT) were scored based on a 0 to 9 scale for stripe rust, as described by Wan et al. [61] and Ren et al. [52]. IT 0–3 were considered resistant, IT 4–6 were intermediate, and IT 7–9 were susceptible.

### 4.4. Physiological Measurement of the Stay-Green Trait

The chlorophyll content, activities of SOD, and CAT, and content of accumulated MDA in the FL, SL, and TL were measured during the process of leaf senescence after anthesis.

The chlorophyll contents of 10 plants from the center of each plot were determined. At 0 DAF (stage I, 0 days after flowering), 7 DAF (stage II), 14 DAF (stage III), 21 DAF (stage IV), 28 DAF (stage V), 35 DAF (stage VI), and 42 DAF (stage VII), the chlorophyll contents of RT843-5 and MY11 were measured using a SPAD-502 chlorophyll meter (Minolta, Milton Keynes, UK) [62]. For each leaf, 10 measurements were taken across the whole leaf blade, with the mean value serving as a representative measurement of the whole leaf blade.

The activities of SOD and CAT and the content of MDA were also measured at the seven stages after anthesis in RT843-5 and MY11 leaves collected from the center part of each plot in the field as described [63,64].

The Pn in the FL was measured using a red-blue light source photometer (Li-6400-02B, Li-Cor, Lincoln, NE, USA) at the seven stages, respectively [33]. Three RT843-5 and three MY11 plants were randomly chosen and marked for measurement. The measurements were taken in the same position and area of the FL. At every sampling date, the mean of three measurements per plant was used to represent the plant phenotypic value, and the average value of three plants was used for the comparisons among genotypes.

### 4.5. Field Experiments

All plants were grown on the experimental farm of the Qionglai Research Station of Sichuan Agricultural University in Southwest China. Three replications with randomized complete blocks were used in the field experiments. The farm experiment was carried out using standard wheat cultivation procedures on the Chengdu Plain. The plants were sown at a density of 160 seedlings/m^2^ in 3 m long plots, each with four rows spaced 25 cm apart. These plots also provided the plants utilized to determine chlorophyll and MDA contents, enzyme activities, and net photosynthetic rates. The NS, KN, TKW, AGB, HI, and yield were determined according to Ren et al. [1,18]. Fungicide was applied to control diseases and pests according to the method by Ren et al. [1,18].

### 4.6. Index Calculation and Statistical Analysis

To reveal the senescence process of the wheat leaf, the SGI, SAI, CAI, MCI, and NPI were calculated as follows: Index = VI/V0 (VI = the measured value on the measurement day after anthesis, V0 = the measured value at 0 days after anthesis) [65,66]. These retention indices depict the visual dynamic variations in the stay-green trait from the filling stage until the dough stage of wheat.

Analysis of variance was performed on the data for each characteristic. The least significant differences (LSD) of mean comparisons were estimated using Sigmaplot 2001 software (SPSS Inc., Chicago, IL, USA). The figures were drawn by TBtools (https://github.com/CJ-Chen/TBtools (accessed on 1 January 2021)), MEGA (https://www.mega.com/ (accessed on 1 January 2020)), and iTOL (http://itol.embl.de/ (accessed on 1 October 2020)).

## Figures and Tables

**Table 1:** Responses to different stripe rust races/isolates of RT843-5.

Translocation Lines and Controls	Chromosome Type	*Pst* Pathotypes and Isolates								
		CYR32	CYR33	CYR34	SY3	SY4	SY5	SY7	HY8	In the Field
RT843-5	T1RS.1BL	0	0	0	0	0	0	0	0	3
CN11	T1RS.1BL	6	6	8	8	8	0	0	6	7
MY11	1B	8	8	8	8	8	8	6	0	9

Infection type: 0: no visible symptoms; 1: necrotic flecks; 2: necrotic areas without sporulation; 3: necrotic and chlorotic areas with restricted sporulation; 4–6: moderate uredinia with necrosis and chlorosis; 7–8: abundant uredinia with chlorosis; 9: abundant uredinia without chlorosis. CN11: Chuannong11; MY11: Miangyang11, the wheat parent of RT843-5.

**Table 2:** Resistance segregation to *Pst* in the backcross RT843-5 × MY11.

Cross		*Pst* Mixture			
	Resistant	Segregation	Susceptible	Ratio	χ^2^
(RT843 × MY11) Testcross	217	-	201	1:1	0.62
(RT843 × MY11) F_2_	224		67	3:1	0.60
(RT843 × MY11) F_3_	64	160	67	1:2:1	2.95

**Table 3:** Analysis of agronomic traits of RT843-5.

Lines	Yield (kg/ha)	NS (m-2)	KN (per Spike)	TKW (g)	AGB (kg/ha)	HI (%)
MY11	5271.3 ± 113.1a	321.7 ± 10.3a	43.3 ± 2.2a	44.4 ± 0.4a	13385.5 ± 1084.8ab	46.6 ± 0.2a
RT843-5	5980 ± 109.4b	307.2 ± 6.1a	40.7 ± 0.7a	47.8 ± 0.4b	12901.7 ± 203.3a	46.4 ± 0.2a

NS: number of spikes per square meter NS, KN: kernel number per spike KN, TKW: 1000-kernel weight, AGB: above-ground biomass, HI: harvest index. Values with the same letter in the same column do not differ significantly at *p* < 0.05.

**Table 4:** The sequences of the molecular markers used in this study.

Molecular Markers	Forward Sequence (5′-3′)	Reverse Sequence (5′-3′)
ω-Sec-P	accttcctcatctttgtcct	ccgatgcctataccactact
Gil-B1	gcagacctgtgtcattggtc	gatatagtggcagcaggatacg
Xgwm582	aagcactacgaaaatatgac	tcttaaggggtgttatcata
Iag95	ctctgtggatagttacttgatcga	cctagaacatgcatggctgttaca

## Data Availability

Not applicable.

## Supplementary Materials

The following are available online at https://www.mdpi.com/article/10.3390/ijms23094626/s1.
