# Supplementary material for: Molecular Cytogenetic and Physiological Characterization of a Novel Wheat-Rye T1RS.1BL Translocation Line from Secale cereal L. Weining with Resistance to Stripe Rust and Functional “Stay Green” Trait
Source: Int J Mol Sci. 2022 Apr 21;23(9):4626. doi: 10.3390/ijms23094626 (PMC9102831; doi:10.3390/ijms23094626)
Supplement: Supplementary file 1 [file ijms-23-04626-s001.zip › supplementary materials/Table S5.pdf]

Table S5. The differences of NPI between RT843-5 and MY11 after anthesis

lines	The days after anthesis.						
	0	7	14	21	28	35	42
RT843-5	1	1.021±0.006b	1.014±0.005b	1.001±0.005b	0.985±0.003b	0.981±0.009b	0.752±0.006b
MY11	1	1.001±0.002a	0.974±0.003a	0.889±0.003a	0.643±0.010a	0.228±0.002a	0a

NPI: Net Pn index
