# Supplementary material for: Molecular Cytogenetic and Physiological Characterization of a Novel Wheat-Rye T1RS.1BL Translocation Line from Secale cereal L. Weining with Resistance to Stripe Rust and Functional “Stay Green” Trait
Source: Int J Mol Sci. 2022 Apr 21;23(9):4626. doi: 10.3390/ijms23094626 (PMC9102831; doi:10.3390/ijms23094626)
Supplement: Supplementary file 1 [file ijms-23-04626-s001.zip › supplementary materials/Table S1.pdf]

Table S1. The differences of SGI between RT843-5 and MY11 after anthesis

	DDA (days after anthesis)						
lines	0	7	14	21	28	35	42
Flag leaves (FL)							
RT843-5	1	0.977±0.04a	0.974±0.06a	0.976±0.04b	0.951±0.06b	0.888±0.05b	0.813±0.06b
MY11	1	0.998±0.03a	0.958±0.06a	0.886±0.04a	0.816±0.04a	0.713±0.04a	0.212±0.02a
Second leaves (SL)							
RT843-5	1	1.009±0.065a	0.996±0.064b	0.824±0.047b	0.738±0.050b	0.709±0.049b	0.662±0.036b
MY11	1	1.026±0.042a	0.795±0.053a	0.556±0.050a	0.363±0.026a	0.225±0.012a	0.191±0.025a
Third leaves (TL)							
RT843-5	1	0.967±0.033b	0.912±0.480b	0.729±0.029b	0.536±0.040b	0.454±0.031b	0.401±0.031b
MY11	1	0.841±0.0529a	0.587±0.042a	0.298±0.028a	0.210±0.017a	0.168±0.019a	0.139±0.020a

SGI: Stay green index
