# Supplementary material for: Molecular Cytogenetic and Physiological Characterization of a Novel Wheat-Rye T1RS.1BL Translocation Line from Secale cereal L. Weining with Resistance to Stripe Rust and Functional “Stay Green” Trait
Source: Int J Mol Sci. 2022 Apr 21;23(9):4626. doi: 10.3390/ijms23094626 (PMC9102831; doi:10.3390/ijms23094626)
Supplement: Supplementary file 1 [file ijms-23-04626-s001.zip › supplementary materials/Table S2.pdf]

Table S2. The differences of SAI between RT843-5 and MY11 after anthesis

	DDA (days after anthesis)						
lines	0	7	14	21	28	35	42
FL							
RT843-5	1	0.953±0.012b	0.888±0.210b	0.841±0.023b	0.781±0.021b	0.708±0.013b	0.585±0.012b
MY11	1	0.710±0.038a	0.586±0.032a	0.395±0.025a	0.290±0.009a	0.243±0.008a	0.210±0.010a
SL							
RT843-5	1	0.949±0.010b	0.776±0.013b	0.638±0.008b	0.557±0.015b	0.538±0.007b	0.469±0.014b
MY11	1	0.656±0.014a	0.274±0.012a	0.218±0.011a	0.187±0.009a	0.140±0.008a	0.126±0.007a
TL							
RT843-5	1	0.858±0.036b	0.718±0.029b	0.479±0.011b	0.225±0.020b	0.109±0.005b	0.075±0.006a
MY11	1	0.740±0.016a	0.385±0.015a	0.148±0.004a	0.095±0.006a	0.080±0.006a	0.068±0.002a

SAI: SOD activity index
