# Supplementary material for: Molecular Cytogenetic and Physiological Characterization of a Novel Wheat-Rye T1RS.1BL Translocation Line from Secale cereal L. Weining with Resistance to Stripe Rust and Functional “Stay Green” Trait
Source: Int J Mol Sci. 2022 Apr 21;23(9):4626. doi: 10.3390/ijms23094626 (PMC9102831; doi:10.3390/ijms23094626)
Supplement: Supplementary file 1 [file ijms-23-04626-s001.zip › supplementary materials/Table S3.pdf]

Table S3. The differences of CAI between RT843-5 and MY11 after anthesis

lines	The days after anthesis.						
	0	7	14	21	28	35	42
Flag leaves (FL)							
RT843-5	1	0.704±0.036a	1.195±0.044b	0.945±0.009b	0.783±0.011b	0.752±0.004b	0.651±0.035b
MY11	1	0.683±0.013a	0.593±0.012a	0.585±0.024a	0.526±0.007a	0.458±0.007a	0.299±0.016a
Second leaves (SL)							
RT843-5	1	0.764±0.157b	0.765±0.024b	0.699±0.018b	0.651±0.030b	0.626±0.028b	0.519±0.025b
MY11	1	0.524±0.006a	0.460±0.004a	0.380±0.016a	0.307±0.002a	0.299±0.010a	0.275±0.006a
Third leaves (TL)							
RT843-5	1	0.711±0.020b	0.661±0.019b	0.496±0.150b	0.381±0.016b	0.331±0.145b	0.331±0.014b
MY11	1	0.427±0.019a	0.355±0.006a	0.330±0.052a	0.274±0.003a	0.258±0.005a	0.258±0.005a

CAI: CAT activity index
