# Supplementary material for: Molecular Cytogenetic and Physiological Characterization of a Novel Wheat-Rye T1RS.1BL Translocation Line from Secale cereal L. Weining with Resistance to Stripe Rust and Functional “Stay Green” Trait
Source: Int J Mol Sci. 2022 Apr 21;23(9):4626. doi: 10.3390/ijms23094626 (PMC9102831; doi:10.3390/ijms23094626)
Supplement: Supplementary file 1 [file ijms-23-04626-s001.zip › supplementary materials/Table S4.pdf]

**Table S4.** The differences of MCI between RT843-5 and MY11 after anthesis

| lines | The days after anthesis. | | | | | | |
|---|---|---|---|---|---|---|---|
| | 0 | 7 | 14 | 21 | 28 | 35 | 42 |
| Flag leaves (FL) | | | | | | | |
| RT843-5 | 1 | 1.102±0.018a | 1.318±0.013a | 1.443±0.011a | 1.511±0.011a | 1.688±0.028a | 2.330±0.018a |
| MY11 | 1 | 1.154±0.023a | 1.865±0.043b | 1.973±0.049b | 2.173±0.055b | 2.366±0.057b | 2.627±0.058b |
| Second leaves (SL) | | | | | | | |
| RT843-5 | 1 | 1.117±0.011a | 1.328±0.022a | 1.456±0.030a | 1.651±0.033a | 2.062±0.040a | 2.312±0.019a |
| MY11 | 1 | 1.165±0.010b | 2.064±0.016b | 2.288±0.029b | 2.452±0.014b | 2.527±0.003b | 2.591±0.021b |
| Third leaves (TR) | | | | | | | |
| RT843-5 | 1 | 1.032±0.018a | 1.444±0.012a | 1.658±0.030a | 2.048±0.016a | 2.187±0.027a | 2.344±0.055a |
| MY11 | 1 | 1.020±0.013a | 1.709±0.014b | 2.388±0.007b | 2.429±0.021b | 2.486±0.045b | 2.491±0.036b |

MCI: MDA content index
